# A Qualitative Exploration of Desired mHealth App Mechanisms Related to Daily Life Influences for College Nursing Students

**DOI:** 10.53520/rdhs2022.10441

**Published:** 2022-06-22

**Authors:** Scott Sittig, Caitlyn Hauff, Susan G. Williams, Rebecca J Graves, Sharon Fruh

**Affiliations:** 1 Department of Health Science, University of Louisiana at Lafayette, Lafayette, Louisiana; 2 Departemnt of Health, Kinesiology and Sport, University of South Alabama, Mobile, Alabama; 3 College of Nursing, University of South Alabama, Mobile, Alabama

**Keywords:** Mobile Health, Nurses, Informatics

## Abstract

**Introduction:**

Mobile health (mHealth) apps are digital health tools that allow for the delivery and access to vital health information, support, and encouragement needed to foster positive behavior change. Designing and developing mHealth solutions based on daily life influences for nursing students is imperative to establishing healthier physical and mental health habits.

**Methods:**

Multiple focus groups (n=10) were conducted, and a questionnaire (n=11) was administered to undergraduate students in the professional nursing component. Themed analysis of focus-group data was conducted along with descriptive analysis of the questionnaire.

**Results:**

All participants stated it has been more difficult to maintain a healthy lifestyle since beginning the nursing program. This deterioration can be attributed to three key areas: mental health needs/support, rigor of nursing school, and decline in positive health choices. Participants stated they would use an mHealth app designed specifically for nursing students to combat deterioration of their health.

**Conclusions:**

The results of this study (100% positive response rate) reveal mHealth applications might be a powerful tool in helping nursing students transform their physical and mental health. It appears that if an mHealth application is created with the specific “must-haves” of nursing students then we might experience a positive shift in health behaviors for nursing students, which will hopefully transcend into their careers as nurses.

## Introduction

Nursing represents the largest workforce in healthcare delivery with nearly 4.2 million nurses working in the U.S. as of 2021.^1^ From the first class in nursing education, students are taught to provide care for others. Still, they often receive little preparation to care for themselves, which can lead to deterioration of their own mental and physical health.^2^ Self-care can be described as purposeful attempts of individuals to maintain their own health as well as preventing and managing illnesses.^3^ A recent study related to self-care strategies in nurses indicates there is generally poor compliance with medical recommendations and self-care among nurses.^4^ However, approximately 31% of the nurses in this Polish study engaged in supplement use such as vitamins and minerals to help boost the immune system. Lack of self-care in nursing may also be referred to as “self-endangering” which involves burn-out in nurses’ mental health.^5^ Burn-out is considered a state of emotional exhaustion as well as low energy levels, negative attitudes, reduced accomplishments, and depersonalization.^6^ A recent meta-analysis on the prevalence of burnout in nurses endorsed that burn-out can come from overload and stress in the work environment, being a single individual, having professional seniority and being a male, and experiencing aggression at work.^7^ If these issues are not addressed by the health-care professional experiencing them or by managers in the workplace, burn out will continue to exacerbate a growing problem.^8^

Stress on individuals while in nursing school is often overlooked and not easily understood. In a recent study, researchers examined perceived stress and other measures of well-being including self-compassion, life satisfaction, and happiness while in nursing school.^9^ Results indicated 56.6% of students’ health had declined since starting nursing school. However, those students who exercised or practiced meditation had higher levels of well-being and lower levels of stress. As discussed by Moore and DeHaan,^10^ often, students who are entering a nursing school program do not anticipate the stress related to the nursing curriculum, clinical courses, and the fear and anxiety associated with taking care of patients and handling the everyday rigor of passing each course. Tips provided by these students include getting proper sleep, belonging to a study group, taking breaks, feeding your body and brain, and participating in encouraging resources offered such as support groups and Bible studies^10^.

One tool that can increase self-care engagement in nursing students is a mobile health (mHealth) application (app). mHealth apps are convenient digital health tools that allow for the delivery and access to vital health information, support, and encouragement needed to foster positive behavior change.^11^ In addition, mHealth apps allow for the delivery of health information at the correct time which is vital to establishing steps toward positive behavior change.^11^ The transition to using mHealth apps to encourage positive lifestyle choices for nursing students should be easier than traditional populations as many nursing students already use mHealth apps to access resources (i.e., medication dosage, patient notes, etc.) during clinical rotations.^12^ However, although nursing students utilize apps for clinical rotations very few utilize mHealth apps for their personal physical and mental healthcare.^13^

The design of mHealth apps often lacks the incorporation and evaluation of user needs assessment.^14^ In order to improve engagement of and sustainable positive outcomes for the mHealth consumer (i.e., nursing students), researchers need to engage the designated end-user (i.e., nursing students) in the creation of an mHealth app which enables them to become more effective self-managers of their daily health and wellness habits.^15,16^

With this in mind, the research team conducted focus group sessions with and administered a questionnaire to undergraduate nursing students in an effort to gain a better understanding of what features nursing students would most like to see in a mHealth app for their personal and mental health use. Specifically, the researchers aimed to understand what elements of daily life influences nursing students’ overall health while in nursing school and mHealth app mechanisms they would utilize. Understanding these components will hopefully inform developers on what is needed in a mHealth app to promote sustainable healthy habits for nursing students and, ultimately, engagement in self-care. Additionally, improving the health of nursing students could carry over to better health outcomes for future nurses and the patients for whom they provide care.

## Scientific Methods

The present study was approved by the research team’s University Institutional Review Board and took place as part of a larger study focusing on nursing student physical and mental health. Five virtual focus groups were conducted with undergraduate nursing students enrolled in the professional nursing component from a university in the Southeast United States. The focus group sessions were conducted on April 13, 2020 with one participant, April 16, 2020 with two participants, April 20, 2020 with three participants, April 22, 2020, with one participant, and May 11, 2020 with three participants.

Eleven participants (including the 10 focus group participants; see Table 1) also completed a questionnaire which consisted of 21 questions. The questionnaire was administered prior to the focus group sessions with one student deciding not to participate in the focus group session. The questionnaire focused on lifestyle questions, smartphone use, mHealth app usage, along with demographic questions such as gender, race, and age (see Table 2).

Participants were recruited through email which was sent to all undergraduate nursing students in the professional component. Each participant completed an informed consent prior to the focus group sessions and were remunerated a $25 e-gift card for attending the focus group session. The incentives were provided to them at the completion of the focus group session.

### Participants

The research team conducted a series of focus group sessions and a social demographic survey to gain a better understanding of what features nursing students would most like to see in an mHealth app. The participants who completed the questionnaire were all female (n=11) and were registered as Bachelor of Science students in Nursing (BSN). Participants ranged in age from 22–42 and were Caucasian (n=7) or African American (n=4). Most were undergraduate Seniors (n=7), and the remaining were Juniors (n=4).

### Protocol

On average, each focus group session lasted 1.5 hours in duration and was conducted utilizing the Zoom Web Conferencing Tool.^17^ The two moderators for the focus group sessions were faculty members from Health, Kinesiology, and Sport and Information Systems and Technology and have conducted qualitative research in the past. The participants were informed that the faculty members conducting the interviews were not members of the nursing faculty in order to ease any concerns about coercion and to allow for open dialogue and discussion. In addition, the participants were informed that upon conclusion of the transcription of the focus group sessions the recordings would be destroyed, and their information would remain anonymous. The questionnaire was designed and delivered utilizing Qualtrics. The students who were interested in participating in the focus groups received an email which included the link to the secure Qualtrics survey.

### Statistical Analysis

**Figure 1.**
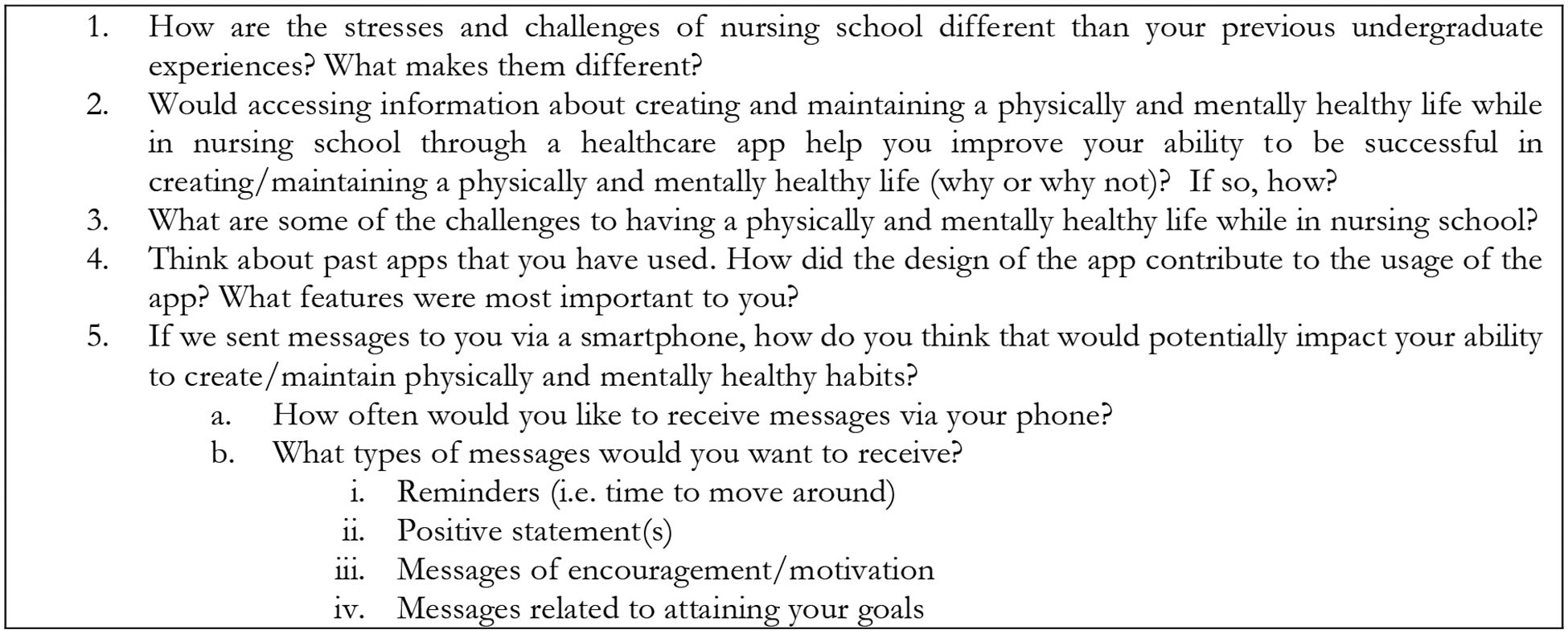
Sample Focus Group Questions

The goal of the focus group sessions was to gain a better understanding of features nursing students would most like in an mHealth app and what were the daily life influences leading to deteriorating physical and mental health while they were in nursing school. The researchers utilized a semi-structured focus group question methodology to stimulate open discussions based on the questions that were selected.^18^ Examples of the type of questions utilized in the focus group sessions can be found in Figure 1. The focus group sessions were recorded through Zoom and then transcribed by a member of the research team. The transcription was then reviewed by two additional members of the research team to evaluate for completeness. At this point NVivo software was utilized to identify key concepts, themes, and repeating patterns. The researchers evaluated the focus group sessions independently and then met to discuss their findings and come to a consensus on emerging themes from the data. In addition, descriptive statistics were generated from the questionnaire (see Table 2) along with qualitative analysis for the open-ended questions. The open-ended question responses were grouped together to determine themes and frequencies were reported.

## Results

Emergent themes revealed the following content areas as “must-haves” in a nursing student-specific app: time management (e.g., calendars, to-do lists, checklists); stress management; physical activity; nutrition (e.g., budget-friendly, quick meals); hydration; and support. The nursing students also revealed the following design features would be necessary for sustainable mHealth app use: personalized; customizable; receive frequent notifications and reminders; interactive; syncs to wearable devices (e.g., FitBit); syncs to phone calendar; contains community platforms for social engagement; and adaptable based on progress and changes to schedule. It is important to note that nursing students stated they desire an mHealth app which would meet their specific needs, but the app must meet most of the above criteria to garner their continual use and engagement. Many of the mHealth apps currently used by nursing students do not meet all these needs at once, so there is reason to believe that developing a comprehensive mHealth app for this population could aid significantly in improving their physical and mental health.

## Themes

### Mental Health Needs and Needed Support.

The participants continually stressed they have struggled to maintain a healthy mental status and need additional positive mental health support. The following seven categories were aligned with the mental health needs of nursing students and their desires for support in a mobile health app: (1) lack of access to timely mental health counseling, (2) mental health of nursing students carries a stigma, (3) high anxiety and stress levels, (4) need for stress management, (5) lack of sleep, (6) need for encouragement (particularly from professors), and (7) the need for meditation.

### Rigor and Structure of Nursing School.

The following five categories were affiliated with the rigor and structure of nursing school: (1) not prepared for the workload, (2) large amounts of paper due after clinical rotations, (3) high anxiety and stress due to high stake courses, (4) no personal time, and (5) overall health deterioration since enrolled in nursing school.

### Health Changes Since Becoming a Nursing Student.

The following eight categories were aligned with what the participants stated in regards to health changes since entering nursing school: (1) struggle with weight, (2) lack of sleep, (3) time constraints (unable to prioritize family and self-care), (4) poor eating habits, (5) less physical activity, (6) increased stress, (7) awareness to avoid disease, and (8) recognize the need to focus on self but lack time to do it. Participants stated that their overall health has deteriorated since joining nursing school. Participants stated their healthy eating habits have declined with one participant saying: “I have gained 30 pounds since joining nursing school”.

### Desired mHealth App Functionality.

The following ten categories were aligned with the students desires for features within a mobile health app: (1) reachable/attainable goals, (2) connectivity to other smart devices (i.e., watches) and email, (3) personalization, (4) competition amongst their peers, (5) group exercise meet-ups through the app, (6) focus on mental health, (7) positive cues/reminders, (8) journaling feature, (9) meditation/mindfulness features, and (10) information on healthy living (i.e., eating healthy, times to exercise (calendar functionality) and movement reminders).

### Types of Cues/Reminders.

The following six categories were aligned with information the participants provided about cues/reminders: (1) motivational and encouraging messages, (2) reminders at the appropriate time, (3) positive affirmation messages from other nurses, (4) customizable, (5) goal-oriented to keep them on task, and (6) the ability to engage with an e-coach.

### Current Apps Features that are Liked.

Some participants stated that they currently utilize mHealth apps. Although these apps are not geared toward their needs as college nursing students, there are some features the participants currently utilize which they feel could be included in a college nursing student mHealth app. The following six categories were aligned with features of currently utilized apps for which the participants like: (1) tracking of exercise (i.e., steps, heart rate), (2) notifications, (3) connection to other devices and ability to interact with other people, (4) customizable, (5) visually appealing, and (6) real interaction (no computer-generated responses).

## Discussion

College nursing students continue to struggle with the stress and workload of nursing programs^19^ leading to poor physical^19^ and mental health habits^20^ and outcomes.^21^ The focus group results showed that all participants acknowledged it has been more challenging to maintain a healthy lifestyle since starting nursing school. This deterioration can be attributed to three key areas: mental health needs^22^ and support,^23^ the rigor of nursing school,^24^ and decline in positive health choices and outcomes.^25^ One digital health tool that can help support nursing students are mHealth apps; especially those geared toward the specific needs of nursing students. This is supported by the fact that all the participants in this study stated they would be willing to use a healthcare app on their smartphone to help with their mental health concerns (depression, anxiety and/or stress) and to help improve their overall health. The participants discussed the key features of what a nursing student mHealth app should contain. The most important features were: creating attainable goals, connectivity to other smart devices, personalization, competition amongst peers, group exercise meet-ups, mental health, positive cues/reminders, journaling, meditation/mindfulness, and information on health living.

In regard to the features of the mHealth app, the participants focused on cues/reminders as a methodology of interaction with the mHealth app. In particular, they expressed a strong desire to have motivational/encouraging messages along with other positive affirmation messages from other nurses. This type of “want” within a mHealth app coincides with the participants’ statements of their “mental health has deteriorated since nursing school” and that they are seeking non-stigma-based mental health management tools. These results further strengthen the ideology of a one-size-fits-all approach does not necessarily work as it relates to mHealth apps. Understanding the unique needs of population and their desired mHealth features is essential to creating a sustainable digital health tool which will hopefully foster positive behavior change for current nursing students, who are the nation’s future nurses.

This initial examination of the desires nursing students want within in a tailored mHealth app provides us with foundational knowledge as to how we can work to make an app like this a reality for nursing students. The potential for an app like this is extensive, as this type of app could expand beyond the population of nursing students to other health professionals, including students in related fields (e.g., medicine, physical therapy, occupational therapy) and graduate students. Future research should assess if the same concerns and desires exist in these populations, as well as their enthusiasm about using an mHealth app to alleviate some of the stress they experience as students.

Despite the important findings of this research study, limitations do exist. The sample size for this study was small, with only 10 nursing students engaging in the focus groups. Although we believe that data saturation was met with this small sample, the limited number of participants and the makeup of our participants in terms of their demographics, do not make the results entirely generalizable to the broader population of nursing students. Our sample consisted of students who were juniors and seniors in the program, so their responses may not represent the same experiences as a newer student in the program, or a graduate student. Additionally, while the researchers made the decision to not to have any nursing faculty present in the focus group, it is possible that participants responded in socially desirable ways, so potential subject bias must be considered when reviewing the findings. Lastly, it is important to keep in mind that this data was collected in April and May of 2020, during the beginning of the COVID-19 pandemic. It is possible that the participants included their current experiences with COVID-19 in their responses, which might impact the transferability of our findings to current nursing students who are experiencing the pandemic in different way. While we believe many of their concerns and desires remain the same, future research should explore the current concerns and desires nursing students have in a post-pandemic world.

## Conclusions

The results of this study support that mHealth apps might be a powerful tool to help nursing students improve their physical and mental health; however, a significant challenge faced when using an mHealth app for behavior change in this population is creating an application which is tailored specifically to the unique needs of nursing students. This population experiences high levels of stress and burn-out, and many students who enter this major often express that they are taught how to care for others but not necessarily how to care for themselves. There is a crucial need to find a sustainable intervention to assist the physical and mental self-care needs of nursing students, and it appears that if an mHealth app is created with these specific “must-haves” in mind, nursing student programs could see a positive shift in the health behaviors of their nursing students, which will hopefully transcend into their careers as nurses. Currently, to our knowledge, no mHealth apps exist on the market whose target population is nursing students. Future research should aim to create an mHealth app utilizing a user-centered-design approach and incorporating elements which are desirable to nursing student end-users.

## Figures and Tables

**Table 1.** Participant Demographic Data, (N=11)

Category	Classification	Results
Gender, n	Female	11
	Male	0
Age, M	Years	27
Race, n	African American	4
	Caucasian	7
Classification, n	BSN Junior Year	4
	BSN Senior Year	7

**Table 2.** Participant Questionnaire Response Data (N=11; sample)

Question	Response	Response Frequency
Since starting nursing school, has it been more difficult to maintain a healthy lifestyle (physical and/or mental)?	Yes	11
	No	0
Since starting nursing school which of the following have been the most difficult to maintain in regard to a healthy balance?	Low or well-managed stress	7
	Regular physical exercise	8
	Health dietary consumption	9
	Positive mental stress	8
Which characteristics of nursing school contribute to your inability to maintain a healthy lifestyle?	Clinicals	4
	Work overload	6
	Studying	1
	Time management	6
	Lack of sleep	2
Do you own a smartphone	Yes	11
	No	0
Do you have access to Wi-Fi daily	Yes	11
	No	0
Do you use your smart phone to download mobile health apps to improve YOUR mental and/or physical health?	Yes	7
	No	4
Do you use your smart phone for your personal health reminders)?	Yes	9
	No	2
Would you be willing to use a mobile health app to help you improve your overall health?	Yes	11
	No	0
On how many days per week would you use a smart phone app to help you with physical activity, diet, and overall health promotion?	Days (mean)	6
How many text messages would you like to receive PER DAY to help you stay on track with exercise, diet, sleep, or any other health improvement goals?	Texts per day (mean)	3
How many days PER WEEK would you like to receive text messages that help you track your goals?	Days per week (mean)	5
Would you be willing to use a healthcare app on your smart phone to help you with mental health concerns such as depression, anxiety, or stress?	Yes	11
	No	0
